# Emergency Oral Contraceptive Consultations in Pharmacies in a Rural Setting: An Epidemiological Analysis

**DOI:** 10.1177/0897190020961698

**Published:** 2020-09-29

**Authors:** Emma Pearce, Kate Jolly

**Affiliations:** 1 Murray Learning Centre, Institute of Applied Health Research, University of Birmingham, Birmingham, UK

**Keywords:** emergency contraception, levornorgestrel, ulipristal acetate, pharmacy

## Abstract

**Background:**

Emergency contraception has been available in pharmacies across England since 2001.There is a paucity of evidence describing those women accessing the service, particularly in rural locations, where pharmacies are integral to improving healthcare accessibility.

**Methods:**

Routinely collected data from all pharmacy consultations for emergency contraception in Shropshire, England, were obtained and anonymized for the study period April 1, 2016 to January 31, 2019. Consultations were described by time, age of consultee, rationale for consultation, method dispensed (levonorgestrel or ulipristal acetate), referral for copper intrauterine device fitting, chlamydia screening where appropriate and reason for choosing pharmacy setting. Repeat attenders were also described separately.

**Results:**

3499 consultations occurred during the study period; 39% were aged between 16-20 years, and 52% attended following unprotected sexual intercourse. Levonorgestrel was initially most prescribed, however ulipristal acetate overtook it in 2018. Onward referral for copper intrauterine device and age-appropriate chlamydia screening took place in 3% and 4% of the eligible populations respectively. Women overwhelmingly chose the pharmacy setting owing to its convenience. Repeat attenders tended to be younger than single attenders, but otherwise similar.

**Conclusion:**

Pharmacy-based emergency contraception is an important and well-utilized service in this rural location and continued funding and possible service expansion should be considered.

## Background

Women in England have been accessing over-the-counter oral emergency contraception in pharmacy settings since 2001, increasing their opportunities to take positive steps toward improved reproductive health.^1^ Two types of oral emergency contraception are available in these pharmacies: levonorgestrel and ulipristal acetate. Ulipristal acetate may be taken up to 120 hours following contraceptive failure or unprotected sexual intercourse, whereas levonorgestrel must be taken within 72 hours.^2,3^ Although the most effective method of emergency contraception is insertion of the copper intrauterine device within 120 hours, this requires specialist assessment and skills in a general practice or sexual health setting and cannot be provided in pharmacies.^4,5^ Despite many years of availability, few studies have examined the characteristics of women accessing oral emergency contraception from pharmacies, particularly those in more rural locations.^1,6^

Women living in rural English counties often face specific reproductive health challenges related to service accessibility. Sexual and reproductive health clinics are less likely to be located near to home, and often have shorter opening hours and fewer staff than higher footfall, inner-city clinics. Rural English counties, in comparison to more urban areas, also have poorer access to general practice services, although maintain adequate access to community pharmacies; this effect is independent of levels of social deprivation.^7,8^ Transport links within and between rural areas are often fragmented, and car ownership can be variable, with young people in particular experiencing problems accessing services not located near major public transport hubs or schools.^9^ Assurance of confidential service provision also ranks higher for young people accessing sexual and reproductive health services in rural locations compared to their urban counterparts.^10^ The above considerations impact not only upon the provision of emergency contraception, but also on access to reliable ongoing contraceptive methods, alongside the broader range of sexual and reproductive healthcare.^11,12^

Current public health challenges, both monetary and service pressures, are negatively impacting the provision of services such as emergency contraception access in a variety of locations across England.^13^ In rural counties, where extra challenges are placed in the way of accessing sexual and reproductive healthcare, it is important to understand how services are utilized and by whom, to make a case for retention and improvement of services. Pharmacies are a key location to study as they may provide an alternative route of delivering safe, effective healthcare in rural communities.

The objective of this study is to describe all individuals requesting emergency contraception consultations in pharmacies within Shropshire Local Authority boundaries during the period April 1, 2016 to January 31, 2019.

## Study Method

### Data Source

This study utilizes routinely collected PharmOutcomes data from April 1, 2016 to January 31, 2019.^14^ All emergency contraception consultations recorded on the database during this timeframe were included in the study. Any pharmacy with a pharmacist qualified to provide emergency contraception and under contract with the Local Authority may provide emergency contraception, without charge, to any person who requests it and fits the clinical criteria. PharmOutcomes data must be provided to the Local Authority to ensure adequate reimbursement for the service; data are entered manually by the pharmacist based on self-report of the client at the time of the consultation and reviewed by the Local Authority on a quarterly basis. All fields in the database, excluding free text boxes for further comment and ethnicity of attendee, are mandatory, ensuring receipt of a complete dataset.

Data were accessed in March 2019 by a Shropshire Local Authority data analyst, anonymized by the study author, and stored securely.

### Variables

Time: Consultations are described by financial quarter of attendance, beginning from Quarter Two of 2016 (April to June). As data for the final quarter (Quarter One of 2019) are incomplete, owing to timing of data extraction, all consultations from this period are combined with Quarter Four of 2018, extending this quarter from October 2018 to January 2019.

Age: Age at consultation is displayed in five-year age bands; a separate 2-year category (13-15 years) separates those below the age of consent and therefore subject to Fraser competence assessment as part of the consultation. Subjects were excluded from analyses by age if recorded age was ≤ 12 years or ≥ 60 years, the assumption being that this data point was incorrect. For ease of display, smaller age categories were combined when data were displayed graphically; original results for individual categories can obtained on reasonable request from the corresponding author.

Consultation rationale: Four categories were recorded: unprotected sexual intercourse, condom failure, failure of regular hormonal contraceptive method (combined or progesterone-only pill) or vomiting following emergency contraception usage within 3 hours.

Type of emergency contraception prescribed: Recorded data detailed type of emergency contraception dispensed (levonorgestrel or ulipristal acetate), dosage, clinical comments (free text box) and reasons for not dispensing (free text box).

Onward referral for copper intrauterine device: This field is recorded as “accepted” or “declined.” If accepted, a woman is given contact details of the nearest service provider for copper intrauterine device fitting, and is also offered oral emergency contraception, if clinically appropriate, to provide cover in case of a delay in accessing the service.

National Chlamydia Screening Programme: In England, everyone aged 15 to 24 years should be offered a free chlamydia screening test in a pharmacy setting if attending for emergency contraception or condom purchase/distribution as part of the National Chlamydia Screening Programme.^15^ Data can be entered as “provided,” “not provided” or “age inappropriate.” Owing to the distribution of available data and predetermined age ranges, chlamydia screening is examined in those aged 13-25 years in this study.

Reason for choosing pharmacy setting: Following consultation, women are asked to provide a reason for choosing the pharmacy setting, rather than other locations or online services, for provision of emergency contraception. Six categories were created within the database by the study author: Advice from healthcare professional, advice from friends/family, convenience, advertising, assurance of confidentiality or “other”.

### Data Analysis

Data are displayed graphically and described using percentages. All subjects had a unique anonymized patient number, allowing any consultation linked to a repeat attender to be highlighted and analyzed independently. Repeat and single attendees are compared using the Chi Squared test, with statistical significance set at the 5% level. All analyses were undertaken using Microsoft Excel (Office 365 Version).

### Ethical Approval and Patient and Public Involvement

Ethical approval was obtained from the University of Birmingham (ERN_19-0342). Data is also covered under the Shropshire Local Authority GDPR statement, and written permission to analyze and publish the data was obtained from the Caldicott Guardian in Shropshire Local Authority in February 2019.

This study was designed and completed following stakeholder feedback gathered for a local needs assessment, in which the general public and service providers highlighted anecdotal inequities in access to all forms of contraception within Shropshire.

## Results

**Figure 1.**
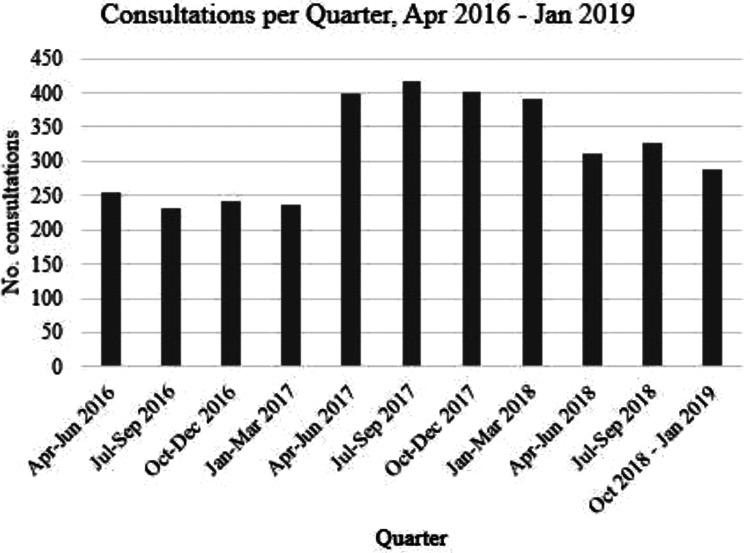
Consultations for emergency contraception in Shropshire pharmacies per quarter, April 2016 to January 2019.

Data were recorded on 3499 emergency contraception consultations during the study period. 3099 individual women attended, and 321 women attended at least twice (721 consultations); this equates to 10% of all individual women accounting for 20.6% of consultations. Consultations per quarter remained at 200-250 from Quarter 2, 2016 to Quarter 1, 2017. In 2017 consultations rose to 330 to 350 per quarter, from Quarter 2, 2017 to Quarter 1, 2018 and then decreased for the remainder of the study period, eventually returning to baseline (Figure 1).

**Figure 2.**
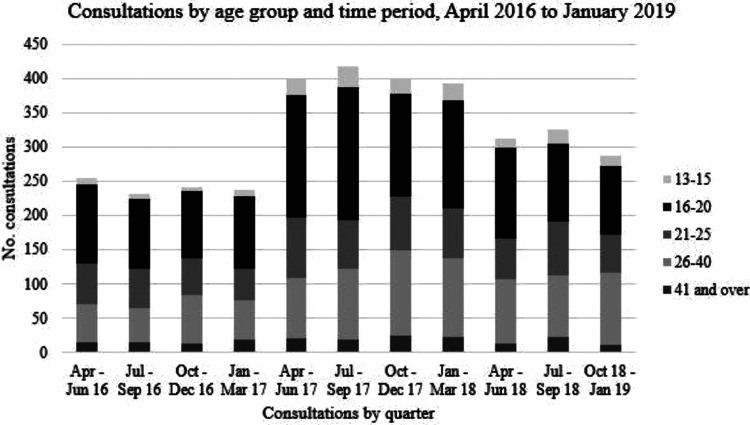
Consultations by age group and time period, April 2016 to January 2019.

Just 2 attendees had recorded ages outside of 13 to 55 years. Sixty-six (1.9%) consultations did not result in emergency contraception dispensing. Commonly identified reasons included clinical suspicion of ectopic pregnancy, delayed presentation, or incorrect presentation. Consultations were noted to be influenced by age. Pharmacy services were most used by those aged 16-20 years, with 39% of all subjects belonging to this group. Consultations decreased rapidly after the age of 20 (Figure 2). Of note, the number of consultations by those aged 13-15 years was similar to the numbers aged 36-40 years and 41-45 years. No significant difference was seen by age group over time. Just over two-thirds (67%) of the total study population were aged between 13-25 years. Of these, 4.7% received a chlamydia screening test during the study period.

**Figure 3.**
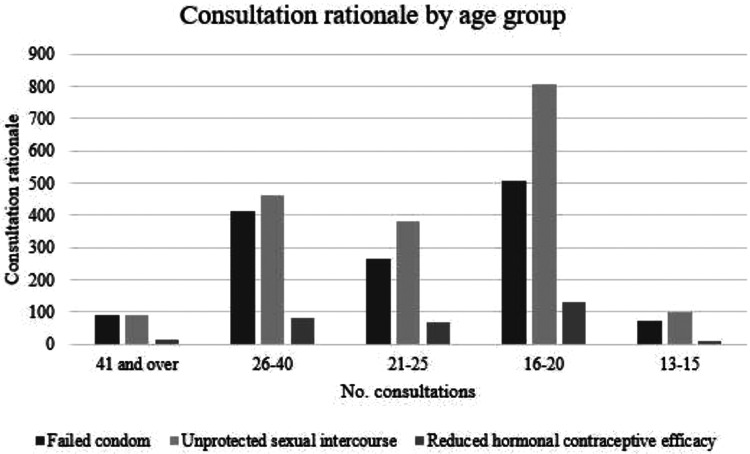
Consultation rationale by age group, April 2016 to January 2019.

Slightly over half of consultations (52.6%) were for unprotected sexual intercourse; 38.4% were for condom failure and 8.8% were for failure of regular hormonal methods. Just 2 consultations (0.02%) were prompted by vomiting following a previous administration of emergency contraception earlier that same day. These trends varied little when adjusted for age (Figure 3).

**Figure 4.**
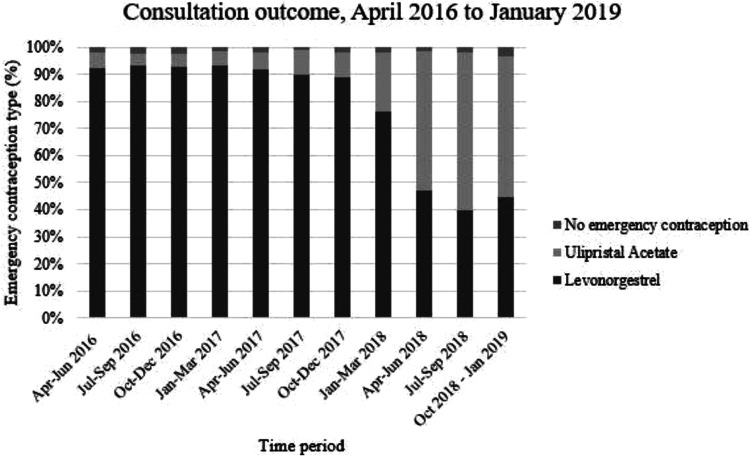
Consultation outcome, April 2016 to January 2019.

Across the whole study period, levonorgestrel was the more commonly supplied emergency contraception method (78.5% consultations). Ulipristal acetate supply remained stable throughout 2016/17, at between 5-10% of supplied items and then increased markedly from the beginning of 2018. From April 2018 onward, ulipristal acetate was the more commonly supplied emergency contraception method, dispensed in over 50% of consultations per quarter (Figure 4). The effect of age on type of emergency contraception was minimal, with around 20% of total consultations resulting in ulipristal acetate supply; this was lowest in those aged 51-55 years (10%) and highest in those aged 36-40 years (26.1%).

Onward referral for copper intrauterine device fitting was accepted in just 3% of all consultations during the study period. Popularity was increased among those aged 31-35 years, with 4.2% of women accepting referral. No women aged 51-55 accepted onward referral for copper intrauterine device fitting. Rates of copper intrauterine device referral remained stable over the course of the study and followed a similar pattern to that of consultation activity overall, with a referral low of 0.78% seen in Quarter 2, 2016, and a high of 5.8% in Quarter 3, 2017. Of the 104 women referred for copper intrauterine device insertion, 91% also received emergency contraception at the time of consultation. The remainder refused on the basis that they did not wish to use any hormonal method of pregnancy prevention.

Convenience was the main reason for choosing the pharmacy over other healthcare settings, as highlighted in 81.4% of consultations. 7.3% of consultations resulted from a healthcare professional recommendation; this was commonly ascribed to a lack of appointment capacity at a general practice/sexual health clinic, or because alternate pharmacies had low stock or no qualified pharmacist on duty. 6.1% of consultations followed recommendations from friends and family, and the remainder of attendances were attributed to viewing advertising for the service (1.1%), feeling reassured about the confidentiality (2%) or other reasons, such as having used that pharmacy before for healthcare reasons (2.1%).

Of the 3099 individual women accessing the pharmacy service during the study period, 321 accessed it on 2 or more occasions. The characteristics of these women are highlighted below and compared to the characteristics of those women attending for a single consultation during the study period (Table 1). A significant difference is seen in the age profile of consultations, with 36.7% aged 16-20 in the single attender group compared to 59.5% in the repeat attender group (p < 0.0001). No significant differences were seen in consultations for unprotected sexual intercourse, acceptance of copper intrauterine device referral or chlamydia screening in the appropriate age group.

**Table 1.** Comparison of Key Characteristics of Single Versus Repeat Consulters.

Characteristic	Single attendance	Repeat attendances
Number of women (% study population)	2778 (89.6%)	321 (10.4%)
Number of consultations (%)	2778 (79.4%)	721 (20.6%)
Consultations aged 16-20 (%)	1020 (36.7%)	429 (59.5%)
Percent unprotected sexual intercourse	52.2%	55.1%
Accepted copper intrauterine device referral (%)	2.7%	3.9%
Chlamydia screening population aged 13-25 (% total population)	1751 (63%)	597 (83%)
Percent screened for chlamydia (eligible population only)	4.7%	5%

## Discussion

Consultations for emergency contraception were commonplace in Shropshire pharmacies between April 2016 and January 2019, and although consultations rose sharply during 2017-18, they returned to baseline by the end of the study period. Based upon 2018 Office for National Statistics figures for women aged 13-55 living within Shropshire Local Authority boundaries, the crude consultation rate for emergency contraception in this study cohort can be calculated at 16 per 1000 women.^16^ Those aged 16-20 represent 39% of all consultations and 53% of consultations across all age groups occurred following an episode of unprotected sexual intercourse. A change in UK-wide prescribing guidance part way through the study period led to ulipristal acetate overtaking levonorgestrel as the most dispensed method of emergency contraception.^4^ Chlamydia screening (in line with the National Chlamydia Screening Programme recommendations) and onward referral for copper intrauterine device was low throughout. 321 women, 10% of all those who consulted for emergency contraception, returned at least twice during the study period. Aside from the age profile, no significant differences between these groups were seen.

This study is the first of its kind to examine patterns in pharmacy-dispensed emergency contraception in a rural location following the change in sexual health funding in England from National Health Service to Local Authority control. This is also one of the first studies to demonstrate the impact of a national change in guidance on the dispensing patterns of ulipristal acetate and levonorgestrel in the pharmacy setting. It has highlighted several important issues, including the high volume of women accessing the service, including those who access it multiple times over a relatively short period of time, poor uptake of the most effective method of emergency contraception and extremely low rates of opportunistic chlamydia screening. By utilizing PharmOutcomes, a large amount of information on a specific population of women has been collected and enables an in-depth examination of a vital component of sexual health service provision that is likely to be replicated in similar counties across England, and other similar health services.

This study is somewhat limited by the data recording method. Changes were made to the database by commissioners in February 2018, meaning that some data, such as previous use of emergency contraception from any provider, ceased to be recorded and could not be reasonably included in this study. Other key characteristics were either not recorded (socioeconomic status, parity) or so poorly recorded they could not be included as a variable in this study (ethnicity). The data is also manually entered and relies upon accurate self-report from the women consulting the pharmacist therefore the data accuracy is likely to vary somewhat depending on how personally sensitive the variable in question is. Finally, by only looking at pharmacies, emergency contraception provision from other service hubs (general practice, accident and emergency departments, sexual health clinics, and, more recently, online services) is not recorded and therefore this study certainly represents an underestimate of the full need for emergency contraception of women residing within the Shropshire Local Authority boundary.

Some of the above findings substantiate previously published studies examining emergency contraception provision in differing populations.^1,3,17^ The age distribution and pattern of repeat use of emergency contraception mirror that of general practice populations seeking emergency contraception prior to the availability of emergency contraception in pharmacies, indicating these women may have changed service provider.^18^ This study confirms current beliefs around pharmacies providing much improved access, and therefore convenience, over other settings from which emergency contraception can be sought.^3,19^ Systematic review evidence supports the finding that young people utilize pharmacies for sexual and reproductive healthcare in large numbers as they find it accessible and acceptable.^20^ Trends in consultation rationale and repeat attendances have also changed very little over time, with Lloyd and Gale noting similar patterns in their 2005 study examining emergency contraception dispensing in a rural part of Yorkshire.^1^ Many studies have previously examined views around pharmacists providing extra services, including chlamydia screening. Thomas et al found that pharmacists believe offering chlamydia screening is an integral part of their job, but in order to avoid offence they tend not to offer it to every eligible women, rather they prefer to make judgements upon the necessity.^21^ This undermines the opportunistic nature of the screening program and the finding is likely to be replicated in our data.^21^

Work has already been undertaken investigating pharmacists’ and women’s views on extending pharmacy-based sexual and reproductive healthcare, including provision of ongoing hormonal contraception at the time of emergency contraception access.^22,23^ These small pilot studies provide promising results but require further refinement, particularly in a rural setting where pharmacists often work alone and may not have capacity to take on the level of work proposed. Economic analysis of these projects in the context of changing public health funding is also required to enable the case to be made for funding if they are found to be effective and acceptable.

## Conclusion

This study highlights the importance of providing a comprehensive emergency contraception service in pharmacies in a rural location in England. It provides evidence for local and national policy makers in sexual and reproductive healthcare to support commissioning decisions and requests for further funding. It also suggests that investing in pharmacies, as a location for sexual and reproductive healthcare may be widely accepted as pharmacies are easily accessible, confidential, and women are often directed to them from other healthcare providers.
